# Doubling Your Payoff: Winning Pain Relief Engages Endogenous Pain Inhibition^1^,^2^,^3^

**DOI:** 10.1523/ENEURO.0029-15.2015

**Published:** 2015-09-17

**Authors:** Susanne Becker, Wiebke Gandhi, Saskia Kwan, Alysha-Karima Ahmed, Petra Schweinhardt

**Affiliations:** 1 Alan Edwards Centre for Research on Pain, McGill University, Montreal, Quebec H3A 0C7, Canada; 2 Faculty of Dentistry, McGill University, Montreal, Quebec H3A 0C7, Canada; 3 Department of Cognitive and Clinical Neuroscience, Central Institute of Mental Health, Medical Faculty Mannheim, Heidelberg University, 68159 Mannheim, Germany; 4 Department of Neurology and Neurosurgery, Faculty of Medicine, McGill University, Montreal, Quebec H3A 0C7, Canada

**Keywords:** motivation, pain modulation, perception, relief, reward

## Abstract

When in pain, pain relief is much sought after, particularly for individuals with chronic pain. In analogy to augmentation of the hedonic experience (“liking”) of a reward by the motivation to obtain a reward (“wanting”), the seeking of pain relief in a motivated state might increase the experience of pain relief when obtained. We tested this hypothesis in a psychophysical experiment in healthy human subjects, by assessing potential pain-inhibitory effects of pain relief “won” in a wheel of fortune game compared with pain relief without winning, exploiting the fact that the mere chance of winning induces a motivated state. The results show pain-inhibitory effects of pain relief obtained by winning in behaviorally assessed pain perception and ratings of pain intensity. Further, the higher participants scored on the personality trait novelty seeking, the more pain inhibition was induced. These results provide evidence that pain relief, when obtained in a motivated state, engages endogenous pain-inhibitory systems beyond the pain reduction that underlies the relief in the first place. Consequently, such pain relief might be used to improve behavioral pain therapy, inducing a positive, perhaps self-amplifying feedback loop of reduced pain and improved functionality.

## Significance Statement

When in pain, pain relief is relevant to everyone. For individuals with chronic pain, pain relief can be an all-dominant goal. Although it is clear that pain relief is a fundamental motivator, it is unknown whether pain relief gained in a motivated state alters the perception of the remaining pain. It is demonstrated here that pain relief that is obtained in a motivated state engages endogenous pain inhibition compared with pain relief unrelated to individuals’ behavior. High novelty seeking as a personality trait was associated with more endogenous pain inhibition. This knowledge is highly relevant for pain therapy as it could be used to create a self-sustaining and perhaps self-amplifying positive feedback loop of pain inhibition and improved functionality.

## Introduction

The pleasure of pain relief is known to everyone—satisfying, soothing, and much sought after when one is in pain. Particularly for individuals with chronic pain, pain relief is a major, sometimes all-dominant goal. Such a motivated state (i.e. the seeking of pain relief) might induce a change in the perception of relief when obtained, because the motivation to obtain reward (“wanting”) and the hedonic experience (“liking”) of a reward are closely linked and typically enhance each other (Barbano and Cador, 2006; Sherdell et al., 2012; for review, see Barbano and Cador, 2007). Enhanced motivation depends on opioid release in response to reward, increasing the hedonic properties of the reward, which is incorporated in future anticipatory evaluation of reward (i.e. incentive salience; Smith et al., 2011). In turn, increased dopamine release in states of heightened motivation (for review, see Berridge et al., 2009) probably leads to increased release of endogenous opioids (Morgan and Franklin, 1990), thereby enhancing liking.

The interaction between pain and reward, specifically reward associated with positive stimuli, is conceptualized in the Motivation-Decision Model (Fields, 2007). This model predicts pain inhibition via endogenous opioidergic systems when the motivation to obtain reward is prioritized over pain avoidance. Confirming the model and the interaction between dopaminergic and opioidergic systems, rewards such as food or money have been shown to induce endogenous pain inhibition through opioid release (Dum and Herz, 1984) and to reduce the perceived intensity of painful stimuli (Becker et al., 2013). Outside the laboratory, interactions between pain relief as the offset of a negative stimulus associated with reward (Franklin et al., 2013) and pain might be particularly important because many chronic pain patients can achieve some pain relief by certain behaviors such as a change in body posture or pacing. Despite potentially being more important than positive stimuli such as money or food, pain relief as a reward is not discussed in the Motivation-Decision Model, and it remains unknown whether pain relief gained in a motivated state induces endogenous pain inhibition, thereby augmenting pain relief.

Here, we exploited that the mere chance of winning induces motivated states even with purely random outcomes (Clark et al., 2009; Martinez et al., 2009; Dong et al., 2014). To test potential pain-inhibitory effects of pain relief that are gained by the individual in a motivated state, we compared pain relief “won” in a wheel of fortune game to pain relief that occurred unrelated to participants’ behavior. Further, we tested whether the hypothesized pain inhibition is related to personality traits associated with reward sensitivity, specifically novelty seeking and reward dependence, and inversely related to harm avoidance.

## Material and Methods

### Participants

Thirty-five healthy volunteers (18 female, 17 male; mean age, 23.6 years; SD, 6.0 years) participated in one testing session each. Exclusion criteria were any present or past pain condition, psychiatric disorders, excessive gambling, substance abuse behaviors, alcohol consumption of >100 ml of alcohol per week, tobacco use, regular night shifts, or sleep disorders. Because no comparable studies were available, expected effect sizes could not be estimated, and, accordingly, an a priori sample size calculation could not be performed. We therefore decided a priori to test 40 participants, allowing the finding of small to medium effects (*f* = 0.16 estimated with G*Power version 3.1; Faul et al., 2007; repeated-measures ANOVA with within-subject factors) with a significance level of 0.05, and an assumed power of 80%. Five recruited participants were excluded before commencing the wheel of fortune game because skin sensitization did not develop with the use of capsaicin. The study was approved by the McGill University Institutional Review Board, and informed consent was obtained from all participants according to the revised Declaration of Helsinki (2008).

### Thermal stimulation

While participants were playing a wheel of fortune game (see below), they received heat stimuli using a 27-mm-diameter contact thermode (Contact Heat Evoked Potentials, CHEPS; PATHWAY Pain & Sensory Evaluation System, Medoc Advanced Medical Systems). The baseline temperature was 32°C, the rise rate was 20°C/s, and the return rate was 30°C/s. Thermal stimuli were applied to the inner forearm of participants’ nondominant hand after sensitization of the skin using 0.075% topical capsaicin cream. The cream was applied to a 3 × 3 cm area on the forearm. Capsaicin is the active ingredient of chili pepper that induces heat sensitization by activating temperature-dependent TRPV1 (vanilloid transient receptor potential 1) ion channels (Holzer, 1991). The cream was removed after 20 min (Dirks et al., 2003; Gandhi et al., 2013), and the thermode was applied at the location on the forearm. Capsaicin-induced sensitization of the skin was used to allow for potent pain relief as reward and pain increase as punishment without the risk of skin damage (Gandhi et al., 2013). Participants’ pain thresholds were assessed before the wheel of fortune game. Participants were exposed to stimuli of 30 s duration with target temperatures starting at 35°C and increasing by 1°C for each subsequent stimulus. Participants rated the peak of the perceived pain intensity at the end of the stimulation. If their rating was <130 on the pain rating scale (mildly painful; see below), more stimuli were applied with increasing temperatures by steps of 1°C or 0.5°C, depending on the participant’s rating. In the case of ratings >130, more stimuli were applied, with decreased temperatures resembling a staircase method. The temperature rated consistently at ∼130 on the pain rating scale was used to determine the stimulation intensities for the wheel of fortune game.

### Rating scales

Participants rated the perceived intensity and pleasantness/unpleasantness of the thermal stimuli using two horizontally orientated visual analog scales (VASs). The intensity VAS ranged from 0 (“no sensation”) to 200 (“most intense pain tolerable”), with 100 being the pain threshold. The pleasantness/unpleasantness VAS ranged from −100 (“extremely unpleasant”) to +100 (“extremely pleasant”), with the midpoint qualifying the stimuli as hedonically neutral (Villemure et al., 2003; Becker et al., 2013). These VASs were used to differentiate between nonpainful and painful as well as between pleasant and unpleasant sensations. Before commencing with testing, participants were familiarized with the rating scales to ensure that they used the scales appropriately.

### Wheel of fortune game

**Figure 1.**
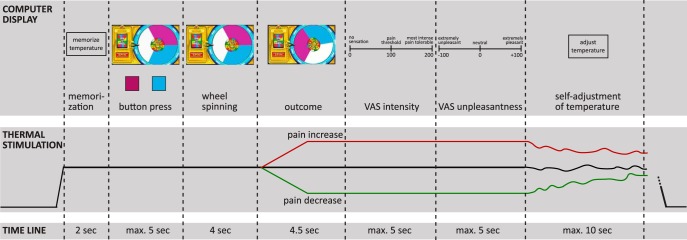
Time line of one test trial of the wheel of fortune game. The green line in the outcome interval indicates pain relief as the outcome of the game, the red line indicates pain increase as the outcome of the game, and the black line indicates no change as the outcome of the game. Thermal stimulation followed the same temperature time course in both the test and the control trials. Instead of playing the game by choosing a color in the button-press interval in the test trials, participants had to press a black button after which the wheel stopped at a random position with no pointer in the control trials. max., Maximum.

A wheel of fortune game, adapted from previous versions (Breiter et al., 2001; Ernst et al., 2004; Becker et al., 2013), was used to provide participants with the possibility of winning pain relief. The game comprised the following two types of trials: the test trials, in which participants played the wheel of fortune game; and the control trials, in which participants did not play the game. In both trial types, thermal stimulation started, and when the target temperature was reached, participants were instructed to memorize the temperature perceived at this moment (interval of 2 s; Fig. 1). After this memorization interval, on a computer screen participants were presented with a wheel of fortune that was divided into three sections of equal size but different color.

In the test trials, participants selected one of two colors by pressing a corresponding button on a keyboard, which started the wheel spinning. When the wheel came to a stop, the color under the pointer determined the outcome. If the wheel landed on the color the participant had selected, the participant won pain relief; if the wheel landed on the color the participant had not selected, the participant lost and received a pain increase; if the wheel landed on the color that could not be chosen (white), the participant neither won nor lost, and the thermal stimulation stayed constant. Both the losing and the no-change outcomes served as control conditions for comparison with the pain relief outcome. The no-change outcome served as a control for unspecific effects for which the difference between test and control trials should not differ for the pain relief and the no-change outcomes, such as distraction. The losing outcome was included because winning has been associated with arousal. Losing is similarly associated with arousal (Sokol-Hessner et al., 2009, 2013) and therefore ensured that any finding regarding winning is not simply caused by arousal. Losing trials also made the game more realistic, which was important to increase participants’ engagement. It was expected that perceived intensities in losing trials would be high, thereby possibly leading to a ceiling effect between test and control conditions for this outcome.

In the control trials, participants could not choose a color on the wheel but had to press a button of unrelated color (black), which started the wheel spinning, as in the test trials. In contrast to the test trials, the wheel displayed in the control trials had no pointer. After the wheel came to a stop, the temperature of the thermode decreased, increased, or stayed the same, just as in the test trials; but because the participant had not selected a color, there was no winning or losing component, and the temperature change occurred unrelated to participants’ behavior. Stimulation intensities in these control trials followed the same course as in the test trials (yoked control) to allow testing specifically for endogenous pain inhibition induced by pain relief that is obtained through winning in a wheel of fortune game.

Unbeknownst to the participants, the outcome of a trial was not related to their color selection because outcomes for each trial occurred in a predetermined, pseudorandom order. This purposefully excluded other processes such as learning and associated meaningful choice behavior, as the aim of the experiments was to test whether pain relief that is won leads to engagement of endogenous pain inhibition compared with pain relief that occurs unrelated to participants’ behavior.

While the outcome temperature of the trial was applied, participants rated the perceived intensity and the pleasantness/unpleasantness of the thermal stimulation using the previously described VASs (Fig. 1). Immediately after these ratings, participants adjusted the stimulation intensity themselves to match the temperature they had memorized at the beginning of the trial to implement a behavioral assessment of pain perception. Participants adjusted the temperature by using a response unit with two buttons, one to increase the temperature and one to decrease the temperature. Self-adjusted temperatures lower than the stimulation intensity at the beginning of the trial indicate sensitization across the trial, while higher temperatures indicate habituation.

Participants played in a total of 18 trials of the wheel of fortune game, 3 trials per condition (test trials: winning, losing, no change; control trials: temperature decrease, temperature increase, no change). Conditions were applied in predetermined pseudorandom order. Each outcome (pain relief, pain increase, no change) occurred with a fixed probability of 1:3.

Pain relief was implemented by a reduction of the stimulation intensity of −7°C, and a pain increase was implemented by a rise of +5°C. The magnitude of these temperature steps was determined and optimized in pilot experiments with the aim of inducing potent pain relief and pain increase. The magnitude of these temperature steps was the same in test and control trials to ensure that the only difference between test and control trials was whether participants played the wheel of fortune game or not.

### Skin conductance measurements

Skin conductance was recorded at the third phalanx of the index and middle finger of the participant’s nondominant hand with Ag-AgCl surface electrodes (Type EL-507) using an MP150 system (BIOPAC Systems Inc.). Skin conductance was sampled at 1000 Hz and high-pass filtered (0.05 Hz). To quantify skin conductance responses (SCRs), the onset to peak amplitude within 1-8 s after the display of the outcome was analyzed. SCRs were averaged across outcomes (pain relief, pain increase, and no change) and trial type (test and control) for each participant. Skin conductance was analyzed using Ledalab version 3.4.6c (Benedek and Kaernbach, 2010).

### Questionnaire and exit interview

The personality traits novelty seeking, harm avoidance, and reward dependence were assessed after the experiment using the Temperament and Character Inventory (TCI; Cloninger, 1987). In addition, an exit interview was performed, asking for the following information: (1) whether participants had difficulties using the VAS or adjusting the temperature; (2) whether participants used a strategy for playing the wheel of fortune; (3) whether participants thought the wheel was more likely to land on one color than another; (4) whether participants thought that the wheel followed a pattern determining the color on which it landed; (5) whether participants were motivated to play the wheel of fortune; and (6) whether participants tried to get as much pain relief as possible while playing the game. Participants first gave yes/no answers and then were asked to specify their answers using open-ended questions.

### Statistical analysis

For the statistical analysis, nine participants were excluded because they did not perceive the thermal stimulation during the wheel of fortune game as being painful (i.e. ratings <100 in the no-change condition), possibly due to the distraction created by playing the game. Before testing the effects of pain relief on the perception of thermal stimuli, it was ensured that the wheel of fortune game did not allow meaningful choice behavior by analyzing the frequencies of choice repetitions after each condition. Frequencies were compared using a repeated-measures ANOVA design with the two within-subjects factors outcome (with the levels pain relief, pain increase, and no change) and trial type (with the levels test and control) by mixed-model procedures. A second repeated-measures ANOVA with the same factors was used to test for possible differences in trial durations because trial durations could vary depending on participants’ speed of responding (Fig. 1).

The effects of pain relief on the perception of thermal stimuli, behaviorally assessed pain perception (adjustment of the temperature) and VAS ratings (perceived intensity and pleasantness/unpleasantness) were analyzed after confirming normality (kurtosis and skewness <1). The onset to peak amplitude of skin conductance responses were squared to correct for non-normality (kurtosis and skewness after correction <1). Behaviorally assessed pain perception, VAS ratings, and squared skin conductance responses were analyzed with a repeated-measures ANOVA design using mixed-model procedures with the factors outcome and trial type. To account for possible ceiling effects in the pain increase outcome, this ANOVA was repeated only for the pain relief and the no-change outcomes. ANOVA analyses were followed by post hoc pairwise comparisons and the calculation of Cohen’s *d* as a measure of effect size (Cohen, 1988) when appropriate.

To test whether the magnitude of pain inhibition due to winning pain relief was related to participants’ personality traits of novelty seeking, harm avoidance, and reward dependence, the differences in behaviorally assessed pain perception, perceived intensity, and pleasantness/unpleasantness between the test and control trials for the pain relief outcome were correlated with the TCI scores.

To assess whether the variables assessed in the exit interview affected the result of the wheel of fortune game, the yes/no answers of the participants were included in the analysis as covariates, calculating separate ANCOVAs with mixed-model procedures for each variable. If the covariate explained a significant amount of variance in the model, it was tested whether this covariate interacted with the factors of interest.

The significance level was set to 5% for all analyses and was Bonferroni corrected for multiple testing. All statistical analyses were performed using PASW Statistics 17 (SPSS Inc.). Table 1 provides a summary of the statistical analyses (rows in the table refer to values referenced by superscript letters in the Results section). Observed power was calculated post hoc with G*Power version 3.1 (Faul et al., 2007).

**Table 1:** Summary of statistical analyses

	Data structure	Type of test	Power
a	Normally distributed	Repeated-measures mixed-model ANOVA, main effect	1
b	Normally distributed	Repeated-measures mixed-model ANOVA, Bonferroni-corrected post hoc comparison	1
c	Normally distributed	Repeated-measures mixed-model ANOVA, Bonferroni-corrected post hoc comparison	1
d	Normally distributed	Repeated-measures mixed-model ANOVA, main effect	0.09
e	Normally distributed	Repeated-measures mixed-model ANOVA, main effect	0.90
f	Normally distributed	Repeated-measures mixed-model ANOVA, interaction	0.05
g	Normally distributed	Repeated-measures mixed-model ANOVA, Bonferroni-corrected post hoc comparison	0.84
h	Normally distributed	Repeated-measures mixed-model ANOVA, Bonferroni-corrected post hoc comparison	0.30
i	Normally distributed	Repeated-measures mixed-model ANOVA, Bonferroni-corrected post hoc comparison	0.84
j	Normally distributed	Repeated-measures mixed-model ANOVA, main effect	0.97
k	Normally distributed	Repeated-measures mixed-model ANOVA, interaction	0.19
l	Normally distributed	Repeated-measures mixed-model ANOVA, Bonferroni-corrected post hoc comparison	1
m	Normally distributed	Repeated-measures mixed-model ANOVA, main effect	1
n	Normally distributed	Repeated-measures mixed-model ANOVA, main effect	0.71
o	Normally distributed	Repeated-measures mixed-model ANOVA, interaction	1
p	Normally distributed	Repeated-measures mixed-model ANOVA, Bonferroni-corrected post hoc comparison	0.30
q	Normally distributed	Repeated-measures mixed-model ANOVA, Bonferroni-corrected post hoc comparison	1
r	Normally distributed	Repeated-measures mixed-model ANOVA, Bonferroni-corrected post hoc comparison	0.05
s	Normally distributed	Repeated-measures mixed-model ANOVA, main effect	1
t	Normally distributed	Repeated-measures mixed-model ANOVA, main effect	1
u	Normally distributed	Repeated-measures mixed-model ANOVA, interaction	1
v	Normally distributed	Repeated-measures mixed-model ANOVA, Bonferroni-corrected post hoc comparison	1
w	Normally distributed	Repeated-measures mixed-model ANOVA, Bonferroni-corrected post hoc comparison	1
x	Normally distributed	Repeated-measures mixed-model ANOVA, Bonferroni-corrected post hoc comparison	0.06
y	Normally distributed	Repeated-measures mixed-model ANOVA, Bonferroni-corrected post hoc comparison	0.06
z	Normally distributed	Repeated-measures mixed-model ANOVA, main effect	=1
aa	Normally distributed	Repeated-measures mixed-model ANOVA, main effect	0.50
ab	Normally distributed	Repeated-measures mixed-model ANOVA, interaction	0.97
ac	Normally distributed	Repeated-measures mixed-model ANOVA, Bonferroni-corrected post hoc comparison	1
ac	Normally distributed	Repeated-measures mixed-model ANOVA, main effect	1
ad	Normally distributed	Repeated-measures mixed-model ANOVA, main effect	1
ae	Normally distributed	Repeated-measures mixed-model ANOVA, interaction	1
af	Normally distributed	Repeated-measures mixed-model ANOVA, Bonferroni-corrected post hoc comparison	1
ag	Normally distributed	Repeated-measures mixed-model ANOVA, Bonferroni-corrected post hoc comparison	1
ah	Normally distributed	Pearson correlation	0.26
ai	Normally distributed	Pearson correlation	0.91
aj	Normally distributed	Pearson correlation	0.19
ak	Normally distributed	Pearson correlation	0.11
al	Normally distributed after transformation	Repeated-measures mixed-model ANOVA, main effect	1
am	Normally distributed after transformation	Repeated-measures mixed-model ANOVA, Bonferroni-corrected post hoc comparison	1
an	Normally distributed after transformation	Repeated-measures mixed-model ANOVA, Bonferroni-corrected post hoc comparison	1
ao	Normally distributed after transformation	Repeated-measures mixed-model ANOVA, Bonferroni-corrected post hoc comparison	0.08
ap	Normally distributed after transformation	Repeated-measures mixed-model ANOVA, main effect	1
aq	Normally distributed after transformation	Repeated-measures mixed-model ANOVA, interaction	1
ar	Normally distributed after transformation	Repeated-measures mixed-model ANOVA, Bonferroni-corrected post hoc comparison	1
as	Normally distributed after transformation	Repeated-measures mixed-model ANOVA, Bonferroni-corrected post hoc comparison	0.71
at	Normally distributed after transformation	Repeated-measures mixed-model ANOVA, Bonferroni-corrected post hoc comparison	0.99
au	Normally distributed	Repeated-measures mixed-model ANOVA with covariate	1

Letters (in the left column) refer to values within the Results section.

## Results

### Effects of pain relief obtained by winning on behaviorally assessed pain perception

**Figure 2.**
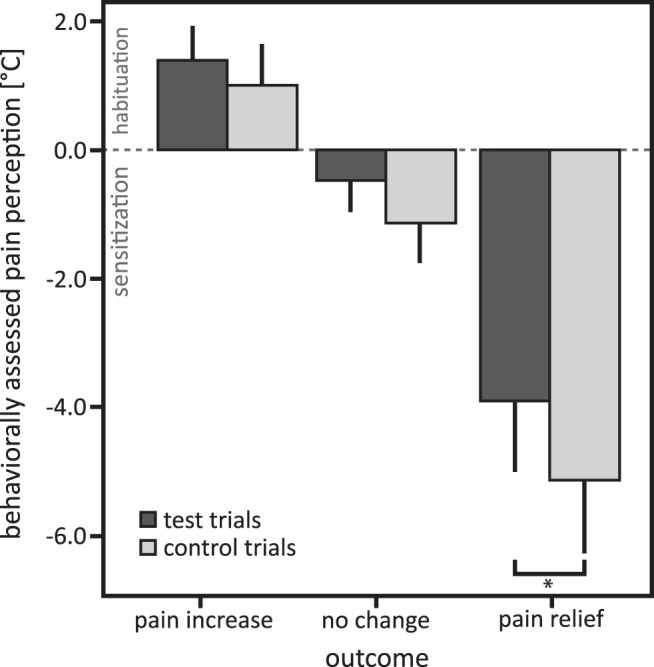
Means and 95% confidence intervals of behaviorally assessed pain perception for test and control trials in the pain relief, pain increase, and no-change outcomes. Negative values indicate pain sensitization relative to the beginning of each trial, and positive values indicate habituation. post hoc comparisons: *p < 0.017, significant after Bonferroni correction for multiple testing.

As expected with ∼20-s-long heat pain stimuli of moderate to high intensity, participants sensitized within trials to the thermal stimulation. In the no-change condition, the self-adjusted temperature was on average 0.8°C lower at the end of the trial compared with the beginning of the trial (mean, −0.80°C; SD, 1.40°C). The self-adjusted temperature was across trial types lower for the pain relief outcome and higher for the pain increase outcome compared with the no-change outcome (Fig. 2; main effect “outcome”: *F*_(25)_ = 162.97, *p* < 0.001^a^; post hoc comparison winning vs no change, *p* < 0.001, Cohen’s *d* = 1.72^b^; losing vs no change, *p* < 0.001, Cohen’s *d* = 1.42^c^; both were significant after Bonferroni correction), probably induced by the temperature decrease and increase in the outcome interval of the wheel of fortune game. Differences in sensitization or habituation across conditions could not be explained by different durations of the trials (mixed-model ANOVA, interaction outcome × trial type: *F*_(150)_ = 0.23, *p* = 0.80^d^; all post hoc comparisons, *p* > 0.25).

However, when compared with pain relief without winning, pain relief obtained by winning resulted in reduced sensitization in response to the thermal stimulation, indicating endogenous inhibition of the nociceptive input and confirming our hypothesis (Fig. 2; main effect trial type: *F*_(25)_ = 8.46, *p* = 0.004^e^; interaction outcome × trial type: *F*_(25)_ = 0.97, *p* > 0.25^f^; post hoc comparison, *p* = 0.007, significant after Bonferroni correction; Cohen’s *d* = 0.47^g^; because the interaction did not reach significance, the post hoc tests were Bonferroni corrected; as both the pain increase and the no-change outcome were designed as control conditions, no interaction was expected). Behaviorally assessed pain perception did not differ for the pain increase (*p* = 0.410^h^) and no-change outcome (*p* = 0.151^i^) between test and control trials. Repeating the analysis without the pain increase outcome to account for possible ceiling effects confirmed the results (main effect trial type: *F*_(75)_ = 7.15, *p* = 0.009^j^; interaction outcome × trial type: *F*_(75)_ = 0.70, *p* > 0.25^k^; post hoc comparisons: pain relief outcome *p* = 0.015, significant after Bonferroni correction; Cohen’s *d* = 2.42^l^; no change, *p* = 0.197).

### Effects of pain relief obtained by winning on pain ratings

**Figure 3.**
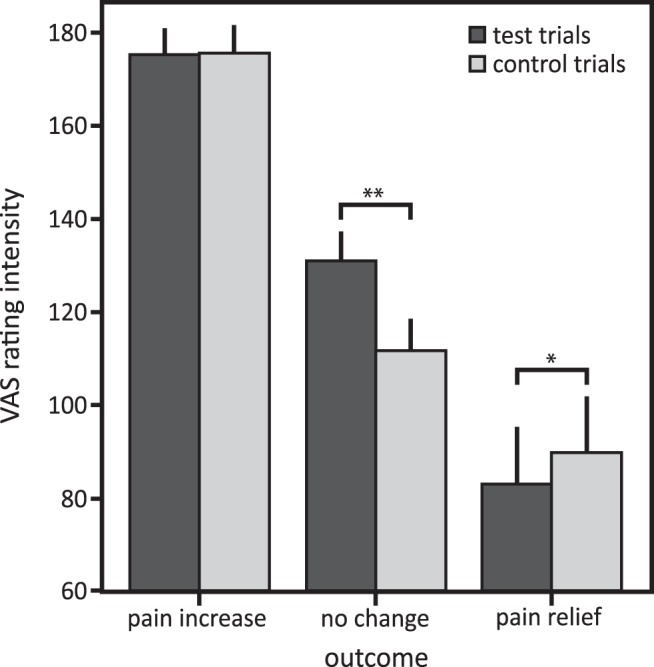
Means and 95% confidence intervals of perceived pain intensity for test and control trials in the pain relief, pain increase, and no-change outcomes. post hoc comparisons: **p < 0.003, *p < 0.017, significant after Bonferroni correction for multiple testing.

Similar to the effects of pain relief obtained by winning on behaviorally assessed pain perception, perceived pain intensity was rated as less intense when pain relief was won compared with the respective control trials without winning (Fig. 3; main effect outcome: *F*_(25)_ = 155.68, *p* < 0.001^m^; main effect trial type: *F*_(25)_ = 5.16, *p* = 0.032^n^; interaction outcome × trial type: *F*_(25)_ = 55.67, *p* < 0.001^°^; post hoc comparison, *p* = 0.011, significant after Bonferroni correction; Cohen’s *d* = 0.23^p^). However, the effect was smaller for subjectively perceived pain intensity compared with behaviorally assessed pain perception (Cohen’s *d* = 0.23 vs Cohen’s *d* = 0.47). In contrast to the behaviorally assessed pain perception, perceived pain intensity differed for the no-change outcome between test and control trials: when participants could choose between two colors on the wheel of fortune (test trials), they perceived the thermal stimulation as more intense when the wheel landed on the color that could not be chosen compared with when participants were not allowed to choose a color (control trials; Fig. 3; post hoc comparison, *p* < 0.001; significant after Bonferroni correction; Cohen’s *d* = 1.13^q^). For the pain increase outcome, ratings of perceived pain intensity did not differ between test and control trials (*p* = 0.932^r^). Repeating the analysis without the pain increase outcome confirmed the results (main effect outcome: *F*_(25)_ = 52.52, *p* < 0.001^s^; main effect trial type: *F*_(25)_ = 8.40, *p* = 0.008^t^; interaction outcome × trial type: *F*_(25)_ = 94.08, *p* < 0.001^u^; post hoc comparisons: pain relief outcome, *p* = 0.011, significant after Bonferroni correction, Cohen’s *d* = 1.14^v^; no change, *p* < 0.001, Cohen’s *d* = 5.75^w^).

No differences in the perceived unpleasantness of the thermal stimulation were found for the pain relief (*p* = 0.759^x^) and pain increase (*p* = 0.791^y^) outcomes between the test and control trials. But similar to the perceived intensity, the stimulation was perceived as more unpleasant when participants were allowed to choose between two colors of the wheel but it landed on the third color (no-change outcome) compared with the respective control trials (main effect outcome: *F*_(25)_ = 294.82, *p* < 0.001^z^; main effect trial type: *F*_(25)_ = 3.46, *p* < 0.001^aa^; interaction outcome × trial type: *F*_(25)_ = 5.55, *p* = 0.005^ab^; post hoc comparison, *p* < 0.001, significant after Bonferroni correction; Cohen’s *d* = 0.88^ac^). The analysis without the pain increase outcome confirmed the results (main effect outcome: *F*_(25)_ = 43.02, *p* < 0.001^ad^; main effect trial type: *F*_(25)_ = 18.25, *p* < 0.001^ae^; interaction outcome × trial type: *F*_(25)_ = 25.62, *p* < 0.001^af^; post hoc comparisons: pain relief outcome, *p* = 0.573; no change, *p* < 0.001, Cohen’s *d* = 4.48^ag^).

Reductions in pain sensitization and reductions in perceived pain intensity due to pain relief obtained by winning were not correlated (*r* = 0.20, *p* = 0.33^ah^), indicating that pain relief that is won may have differential effects on different components of pain processing.

### Association of novelty seeking and pain inhibition by pain relief obtained by winning

**Figure 4.**
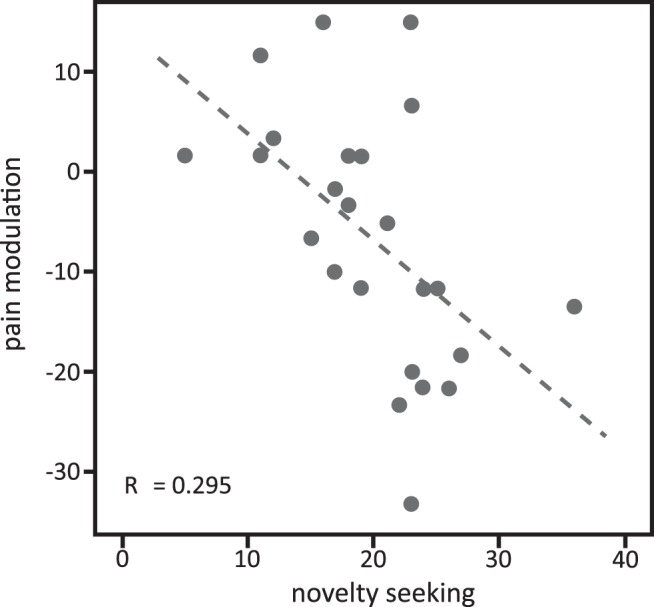
Correlation of participants’ scores on the novelty-seeking subscale of the TCI and pain modulation by pain relief obtained by winning, which was calculated as the difference between intensity ratings in the test minus the control trials of the pain relief outcome.

Participants showed more endogenous pain inhibition by pain relief obtained by winning the more novelty seeking they were: the amount of pain inhibition by pain relief that was won in the test compared with the control trials correlated negatively with novelty seeking assessed with the TCI questionnaire (Fig. 4; *r* = −0.54, *p* = 0.005^ai^). Because pain inhibition by pain relief obtained by winning was calculated as the difference between VAS ratings of perceived intensity in the test and control trials, negative values indicate successful pain inhibition. Novelty seeking was specifically related to induced pain inhibition obtained by winning pain relief and not to the level of the perceived pain in either the test or the control trials, which was demonstrated by computing separate correlations of the pain ratings with the novelty-seeking scores in the test trials (*r* = −0.15, *p* = 0.48^aj^) and control trials (*r* = 0.08, *p* = 0.72^ak^). No correlations were found with harm avoidance and reward dependence.

### Skin conductance responses

Further, as expected for the different thermal stimulation intensities, skin conductance responses differed for the difference outcomes (pain relief, no change, pain increase) of the wheel of fortune, irrespective of the trials type (test, control) indicated by a main effect of outcome (*F*_(62)_ = 7.22, *p* = 0.002^al^). post hoc tests revealed higher skin conductance responses with the pain increase outcome compared with the no-change outcome (*p* = 0.001, significant after Bonferroni correction, Cohen’s *d* = 0.82^am^) and the pain relief outcome (*p* = 0.002, significant after Bonferroni correction, Cohen’s *d* = 1.04^an^; comparison no change – win, *p* = 0.641^ao^).

**Figure 5.**
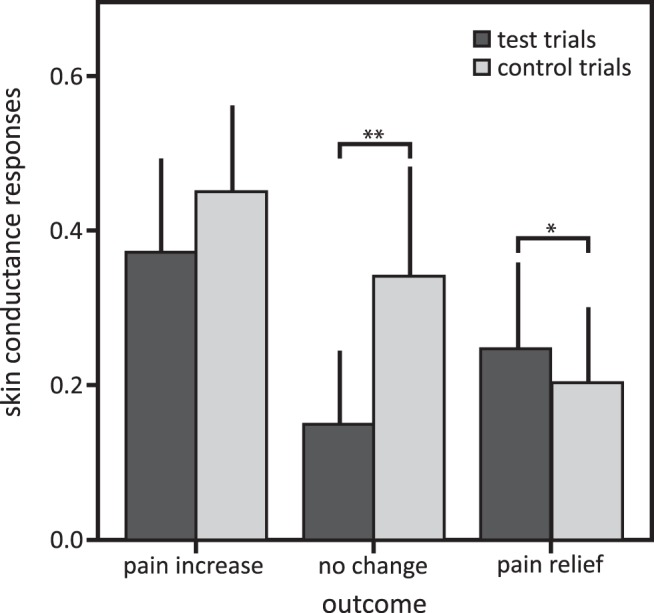
Mean amplitudes and 95% confidence intervals of skin conductance responses in the test and control trials in the pain relief, pain increase, and no-change outcomes. post hoc comparisons: ^t^*p < 0.10; **p < 0.003 after Bonferroni correction for multiple testing.

Skin conductance responses were higher in the test compared with the control trials across outcomes, indicated by a main effect of trial type (*F*_(60)_ = 11.20, *p* = 0.01^ap^). Further, skin conductance responses showed an interaction effect of outcome and trial type (*F*_(54)_ = 6.79, *p* = 0.02^aq^). post hoc comparisons showed a significant difference between test and control trials for the no-change outcome, with higher skin conductance responses in test trials compared with control trials (Fig. 5; post hoc comparison, *p* < 0.001, significant after Bonferroni correction, Cohen’s *d* = 0.96^ar^). In addition, a trend for differences in skin conductance responses for the pain increase outcome between test and control trials was observed, with higher responses in test trials compared with control trials (Fig. 5; post hoc comparison, *p* = 0.07, Cohen’s *d* = 0.42^as^), but no difference for the pain relief outcome (*p* = 0.308^at^). These results indicate that participants were more aroused in the test trials compared with the control trials for the no-change outcome, with a similar tendency for the pain increase outcome, but not for the pain relief outcome.

### Exit interview

The exit interview revealed that four participants had difficulties using the VASs, and one had difficulties memorizing the temperature at the beginning of each trial. Although this variable explained a significant amount of variance as a covariate in an ANCOVA of perceived pain intensities (*F*_(25)_ = 4.04, *p* = 0.046^au^), none of the factors of interest was affected by these difficulties, indicating that the covariate had no direct effects on the effects of pain relief obtained by winning. In addition, the covariate had no effects on the other outcome measures (behaviorally assessed pain perception and perceived unpleasantness). No other variable from the exit interview had any effect on any of the outcome measures.

### Manipulation check

As intended by the design of the wheel of fortune game, the different outcomes of the game in the previous trial had no effect on choice behavior, indicating that reward-dependent learning and meaningful choice behavior were successfully eliminated.

## Discussion

In this study, we show for the first time that pain relief that is gained in a motivated state induces endogenous pain inhibition, thereby augmenting pain relief. Exploiting the fact that the mere chance of winning induces motivated states even with purely random outcomes, we used a wheel of fortune task to induce such a motivated state. These pain-inhibiting effects of pain relief linked to increased motivation were observed in behaviorally assessed pain perception and in ratings of perceived pain intensity. The amount of endogenous pain inhibition was related to the personality trait of novelty seeking: the higher participants scored on novelty seeking, the more their pain was decreased when they won pain relief compared with the control condition.

The present results demonstrate clearly that pain relief, when obtained in a motivated state, engages endogenous pain-inhibitory systems beyond the pain reduction that underlies the relief in the first place. It had been shown previously that monetary reward inhibits pain perception (Becker et al., 2013), but no data existed on pain relief as a reward. Although winning pain relief, as implemented in this study, is not necessarily based on instrumental, contingent behavior, the mere chance of winning induces motivated states, thoughtful decision making, and the illusion of control, even with purely random outcomes (Clark et al., 2009; Martinez et al., 2009; Dong et al., 2014). Also, winning is inherently associated with positive emotions. Because motivational and emotional pain modulation cannot be separated in the present study, mechanisms of affective pain modulation might have contributed to the pain inhibition observed (Villemure et al., 2003; Kenntner-Mabiala et al, 2008; Roy et al., 2008). Nevertheless, pain relief typically occurs in motivated states (i.e. when someone is in pain and is seeking to decrease his or her pain). Particularly in patients with chronic pain, pain relief is sometimes an all-dominant goal. Some pain relief can be achieved by many chronic pain patients, for example, by a change in body posture. Therefore, we posit that pain relief is particularly relevant in natural settings. The motivational component of pain relief shapes future behavior through operant learning (Becker et al., 2011; Navratilova et al., 2012), increasing the likelihood of repeating the behavior that led to the pain relief. Thereby, a positive feedback loop of behavior and pain inhibition that is perhaps self-amplifying might be created.

In the present study, pain inhibition induced by pain relief gained in a motivated state was stronger in the behaviorally assessed pain perception compared with participants’ ratings of perceived intensity. Similarly, operant learning by pain relief as negative reinforcement in behaviorally assessed pain perception, but not in pain ratings, has been found previously (Hölzl et al., 2005; Becker et al., 2011). The effects of pain relief might be better captured by perceptual assessments such as the behavioral assessment of pain perception used here (Kleinböhl et al., 1999) because such behavioral assessments are less influenced by social and cognitive confounds compared with verbal ratings (Cowey, 2004). In addition, it has been shown that even reductions in nociceptive input that are not consciously perceived can act as negative reinforcement (Becker et al., 2012), perhaps indicating that behaviorally assessed pain perception is a more sensitive measure than pain ratings. Further, in contrast to perceived pain intensity, perceived unpleasantness was not modulated when pain relief was obtained in a motivated state. While it is not obvious why such dissociation occurred (we excluded higher variance in the unpleasantness ratings as a possible factor), similar findings have been reported before. For example, it has been reported that attention modulates predominately perceived pain intensity and emotion modulates perceived unpleasantness (Villemure and Bushnell, 2009), but also that emotion modulates both perceived intensity and unpleasantness (Kenntner-Mabiala et al., 2008).

Individuals who are more reactive to reward might benefit more from pain relief in terms of endogenous pain inhibition. This was indicated by the correlation of the personality trait novelty seeking and endogenous pain inhibition: the higher the novelty-seeking scores, the higher the pain inhibition by pain relief that was won. Reward sensitivity, and in particular novelty seeking, has been related to the neurotransmitter dopamine (Leyton et al., 2002; Zald et al., 2008). Thus, the finding that the pain-inhibitory effects of pain relief gained in a motivated state were related to novelty seeking might indicate that dopamine mediated endogenous pain inhibition. In support of this notion, placebo analgesia, in which the anticipation of clinical benefit can be conceptualized as a special case of reward anticipation (de la Fuente-Fernández et al., 2001), has been shown to be associated with higher scores in personality traits related to reward sensitivity, including novelty seeking (Schweinhardt et al., 2009). Also, direct evidence indicates that dopamine mediates the pain-inhibitory effects of monetary reward (Becker et al., 2013). It is conceivable that the motivation to obtain pain relief increases with increasing pain intensity, which in turn is related to increased dopamine release in the basal ganglia (Wood et al., 2007; Scott et al., 2008). Thus, increasing dopamine release might bias an organism more and more toward escape or avoidance behavior to increase the likelihood of pain relief. Obtaining pain relief in such a state of heightened motivation probably increases the release of endogenous opioids (Morgan and Franklin, 1990), augmenting the pain relief and the hedonic experience (liking). The hedonic experience of relief is associated with reward (Franklin et al., 2013), inducing approach behavior and, if applied in a learning context as negative reinforcement, a strengthening of behavior. Nevertheless, relief and reward can be conceptualized as different entities, and relief learning and reward learning appear to be mediated by different neurophysiological mechanisms (for review, see Gerber et al., 2014). Future studies should assess and specify the neurophysiological mechanisms underlying pain inhibition induced by pain relief gained in motivated states.

Participants were more aroused when they played the wheel of fortune game (test trials) compared with the control trials of the game, which is indicated by the higher skin conductance responses. This effect was particularly strong in the no-change condition. A similar trend was observed in the pain increase condition; stronger differential skin conductance responses between test and control trials were possibly precluded by a ceiling effect. In the pain relief condition, skin conductance responses did not differ between test and control trials. This could be explained by a soothing effect of pain relief, reducing arousal, and thereby reducing the difference in arousal between the test and control trials. For the no-change condition, higher arousal in the test trials might explain the higher ratings of perceived pain intensity of the test trials compared with the control trials. As proposed by the “two-factor theory of emotion,” or “Schachter–Singer theory” (Schachter and Singer, 1962; Friedman, 2010), arousal might have been cognitively evaluated, resulting in the interpretation that the higher arousal might be caused by higher pain, leading in turn to higher ratings of perceived pain intensity. No such differential effects of arousal would be expected for implicit behavioral measures (Cowey, 2004; Hölzl et al., 2005); and, indeed, there was no difference between the test and control trials in the no-change condition when pain was behaviorally assessed. An alternative interpretation to the Schachter–Singer theory is that playing the game without winning in the test trials of the no-change condition induced negative emotions, contributing to pain facilitation in these trials (Villemure et al., 2003; Kenntner-Mabiala et al., 2008; Roy et al., 2008).

Endogenous pain inhibition induced by pain relief gained in a motivated state occurred over and above the well known effects of distraction (Duncan et al., 1987; Miron et al., 1989; Villemure and Bushnell, 2009), offset analgesia (Yelle et al., 2008; Martucci et al., 2012), and stimulus controllability (Arntz and Schmidt, 1989; Müller, 2011). Distraction reduces short-term pain to such a degree that it is used in clinical settings (e.g., when children undergo minor medical interventions). Offset analgesia describes the phenomenon that pain reduction is consistently reported as bigger than suggested by the actual change in nociceptive input. Stimulus controllability is associated with reduced pain perception (Arntz and Schmidt, 1989; Müller, 2011), and playing the wheel of fortune game might have induced a feeling of control (Martinez et al., 2009). The effects offset analgesia, and controllability should be present in the test and control trials of the wheel of fortune game and can therefore not have confounded the findings of the present study. Further, attention or distraction effects can neither explain our findings because heightened attention to the thermal stimulation in the test trials, leading to increased pain perception, or distraction by the wheel of fortune, leading to decreased pain perception, would have similarly influenced the pain relief outcome as well as the no-change outcome.

Reinforcement is an important principle that is already used successfully in operant pain therapy. Using reinforcement to improve health behavior and to reduce maladaptive pain behavior results in substantial and long-lasting improved functionality and reduced clinical pain in chronic pain patients (for review, see Flor and Diers, 2007; Gatzounis et al., 2012). Based on the influential work by W.E. Fordyce (Main et al., 2015), positive reinforcement based on social interaction (e.g., verbal feedback or attention) is applied in operant pain therapy. Using pain relief as a negative reinforcement might be of particular benefit in this context because pain relief is a prominent and fundamental motivator for chronic pain patients. As discussed above, pain relief occurs frequently in chronic pain patients, although such relief might be incomplete. Importantly, even if reductions in nociceptive input are very small and possibly below the discrimination threshold (i.e. they cannot be reported), they can shape future behavior through their rewarding properties (Becker et al., 2008). Further, it has been shown that after partial pain relief even moderate pain can be perceived as pleasurable, demonstrating the strong motivational and emotional components of reduced pain (Leknes et al., 2013). Using pain relief in operant pain therapy could create a self-sustaining and perhaps self-amplifying positive feedback loop of pain inhibition and improved functionality, possibly enhancing the effectiveness operant pain therapy.

In summary, our results indicate that pain relief gained in a motivated state induces endogenous pain inhibition and that the amount of this pain inhibition depends on an individual’s degree of novelty seeking. These results highlight the notion that pain relief is a fundamental motivator that can modulate our pain perception. Surprisingly, in clinical contexts, pain relief is commonly viewed as a simple reduction in perceived pain intensity (Farrar et al., 2001). Consequently, clinical trials typically measure only reductions in perceived pain intensity (Dahan et al., 2011; Martini et al., 2013), neglecting important factors; regaining functionality and improving quality of life is often more important for chronic pain patients and, at least partially, are independent of an actual change in pain magnitude. To further expand the present findings and to allow their implementation in pain therapy, future studies should investigate whether chronic pain patients show similar responses to pain relief obtained in a motivated state.

## Synthesis

The decision was a result of the Reviewing Editor Philippe Tobler and the peer reviewers coming together and discussing their recommendations until a consensus was reached. A fact-based synthesis statement explaining their decision and outlining what is needed to prepare a revision is listed below.

Overall, the reviewers have found merit in the design of your study and its potential insights into pain relief. However, in its present form, the study does not sufficiently advance the field. Moreover, the reviewers raise several issues that require clarification.

Major points

First, use of the term "pain relief" needs to be clarified. The paradigm which is outlined in Figure 1, realizes a pain decrease, a pain increase and a pain stable condition, and in addition a fortune wheel and no fortune wheel condition. These conditions, however, are not labelled in the result figures. Here, the authors talk about loosing, winning, and no change conditions, which actually does not hold true since there were no such conditions during the control trials. The winning condition actually is the pain decrease condition, and pain decrease implies pain relief. Instead, the authors use the term "pain relief" only for the condition "pain decrease after winning". However, why is pain decrease in the control trials not experienced as pain relief?

Second, the authors should consistently distinguish "reward" from "negative reinforcement" whenever appropriate. Related to this and to the first point, it would be useful to clarify whether the main difference between the wheel of fortune condition and the control condition is that of instrumental conditioning (even if the control over the outcomes by the subjects' responses is only alleged - psychologically it should still have played a role) and Pavlovian conditioning. By extension, one would wonder why (the definition of) pain relief should be restricted to the former. In any case, "reward" and possibly "pain relief" seem(s) to be the wrong term(s) to distinguish the two conditions.

Third, the authors discuss the possibility of an unconscious pain relief. How is this possible? Can we have unconscious pain and even unconscious pain relief?

Fourth, the authors neglect studies conceptualizing pain relief differentially (summarised e.g. by Gerber et al., 2014, Pain-relief learning in flies, rats, and man: Basic research and applied perspectives, Learning and Memory). Please discuss how the study's results and interpretation fit to major theories in the field. In particular, you may want to distinguish motivational inhibition (Konorskian and Solomon-Corbit opponency) from pain inhibition per se (reducing also perception, for instance via descending modulation). This would help clarify how the results relate to animal studies.

Fifth, the authors should clarify the difference between pain rating and motivation (i.e. relief liking and wanting) in the introduction. What does the MD model have to say about it? (This is also relevant to the discussion about dopamine)

Sixth, is it really fair to call the effect an intrinsic reward, because in psychology, this is normally used to refer to evolutionarily shaped incentives such as play, exploration, novelty seeking etc.?

Minor points

The name of the institution which approved the study should be specified.

Does the loss negative result rule out controllability relief? If so, the authors may want to perform an interaction analysis to show it?

Does the study have any implications for the site of inhibition (cortical/spinal)?

It should be specified by how much the probabilities that were actually realized in the experiment differed from the stated probabilities of 1/3.

The authors should explain how sensitization (e.g. line 154) differs from pain relief.

It should be clarified whether the relation of pain-inhibition with novelty seeking arises primarily from test trials or control trials by computing separate correlations.

Point-by-point response to the reviewer's comments for eN-NWR-0029-15

We thank the reviewers for the careful review of our manuscript and have revised the manuscript based on their suggestions. We think that we were able to address all queries and that the manuscript has thereby been considerably improved. Please find our responses to each reviewer comment below. In addition, we have highlighted the changes in the manuscript.

Major points

Query 1: First, use of the term "pain relief" needs to be clarified. The paradigm which is outlined in Figure 1, realizes a pain decrease, a pain increase and a pain stable condition, and in addition a fortune wheel and no fortune wheel condition. These conditions, however, are not labelled in the result figures. Here, the authors talk about losing, winning, and no change conditions, which actually does not hold true since there were no such conditions during the control trials. The winning condition actually is the pain decrease condition, and pain decrease implies pain relief. Instead, the authors use the term "pain relief" only for the condition "pain decrease after winning". However, why is pain decrease in the control trials not experienced as pain relief?

Please see our response to Query 2.

Query 2: Second, the authors should consistently distinguish "reward" from "negative reinforcement" whenever appropriate. Related to this and to the first point, it would be useful to clarify whether the main difference between the wheel of fortune condition and the control condition is that of instrumental conditioning (even if the control over the outcomes by the subjects' responses is only alleged --‐ psychologically it should still have played a role) and Pavlovian conditioning. By extension, one would wonder why (the definition of) pain relief should be restricted to the former. In any case, "reward" and possibly "pain relief" seem(s) to be the wrong term(s) to distinguish the two conditions.

Response: We agree with the reviewer that the term "pain relief" does not distinguish the conditions implemented in our study well and that we did not use the term "reward" in an unambiguous way. Pain relief, i.e. a decrease of nociceptive input, occurred indeed in the test as well as in the control conditions. The difference between the two conditions is that participants only received pain relief in the test condition when they won in the wheel of fortune game. Based on studies on gambling (e.g. Clark et al., 2009; Martinez et al., 2009; Dong et al., 2014), we presume that the possibility to win in the test condition induced a motivated state, which in turn resulted in the observed pain inhibition in the test condition compared to the control condition. In contrast, pain relief in the passive condition (without playing the game) was not related to participants' behavior. Because outcomes (pain relief, pain increase, or no change in pain) were administered in the control condition pseudorandomly with no relation to the displayed colors (and the wheel of fortune was presented without a marker at the 9 o'clock position) this condition does not resemble a Pavlovian conditioning procedure.

In the test condition, winning pain relief was related to participants' behavior, a pre--‐requisite for instrumental conditioning. However, the test condition did not allow for strengthening of a behavior (a key component of reinforcement) because participants played a wheel of fortune, in which the outcomes were dependent on the participants' luck (at least from the perspective of the participant).

We therefore do not think that the pain reductions linked to winning should be conceptualized as negative reinforcement.

Based on the reviewer's comments, we have revised the entire text and the figure legends in order to differentiate pain relief linked to winning from pain relief without winning and we carefully reviewed the usage of the term 'reward'. In particular, we revised the introduction highlighting that we expected endogenous pain inhibition to be induced by pain relief when gained in a motivated stated compared to situations when getting pain relief is unrelated to behavior.

Query 3: Third, the authors discuss the possibility of an unconscious pain relief. How is this possible? Can we have unconscious pain and even unconscious pain relief?

Response: We assume that the comment refers to the statement "...it has been shown that pain relief can act as reward even if reductions in nociceptive input are not consciously perceived.". A study by Becker et al., (2012) showed that reductions in nociceptive input below the discrimination threshold result in changes of participants' behavior through operant learning (operationalized as negative reinforcement) . Thus, changes in nociceptive input can be processed unconsciously. Nevertheless, pain is defined as an 'experience' (Definition of pain by the International Association of the Study of Pain, IASP: "An unpleasant sensory and emotional experience associated with actual or potential tissue damage, or described in terms of such damage." Loeser and Treede, 2008) and thus, cannot be unconscious. To avoid any potential ambiguity, we rephrased the above--‐mentioned statement to read "it has been shown that even reductions in nociceptive input that are not consciously perceived can act as negative reinforcement" (page 18, line 392--‐394).

Query 4: Fourth, the authors neglect studies conceptualizing pain relief differentially (summarised e.g. by Gerber et al., 2014, Pain--‐relief learning in flies, rats, and man: Basic research and applied perspectives, Learning and Memory). Please discuss how the study's results and interpretation fit to major theories in the field. In particular, you may want to distinguish motivational inhibition (Konorskian and Solomon--‐ Corbit opponency) from pain inhibition per se (reducing also perception, for instance via descending modulation). This would help clarify how the results relate to animal studies.

Response: As pointed out by the reviewer, pain relief can be conceptualized differently, i.e. not as a reward ("onset of something good"; Gerber et al., 2014), but as a separate entity referring specifically to the acute effects of punishment offset ("offset of something bad"; Gerber et al, 2014). As summarized in the review by Gerber et al. (2014), relief learning occurs when behavior gets associated with the cessation of something aversive, particularly pain, but not with the prior onset of this aversive stimulus.

The studies summarized by Gerber et al. highlight that it is potentially important to differentiate between reward learning and relief learning because they might be mediated via different neurophysiological mechanisms. Albeit the experimental design was chosen to prevent learning in terms of forming explicit response--‐stimulus associations, it might be that reward and relief are similarly distinguishable on a neurophysiological level. We therefore explicitly note that different neurophysiological mechanisms might underlie reward and relief (learning; page 19, lines 418--‐420). Our finding that pain relief related to subjects' motivated behavior induced decreased pain sensations, most likely via the engagement of endogenous pain inhibitory systems, clearly indicates that perception was affected. We did not measure motivation and can therefore not comment on potential effects on motivation or motivational inhibition.

Query 5: Fifth, the authors should clarify the difference between pain rating and motivation (i.e. relief liking and wanting) in the introduction. What does the MD model have to say about it? (This is also relevant to the discussion about dopamine)

Response: We assume that the reviewer refers to the pain ratings as an indicator of liking (relief liking in our study) and motivation as an indicator of wanting as two separable components of reward processing. Pain ratings indicate subjectively perceived pain levels and therefore, the difference in ratings between the test and control trials could be viewed as an indicator of relief liking. As pointed out in the response to the previous comment, we did not measure participants' motivation. But the test condition in which participants played the wheel of fortune game promoted motivated behavior and resulted in endogenous pain inhibition relative to the control condition. Thus, using the terminology of liking and wanting, the 'wanting' induced an increase in (relief) liking, which might induce (in other settings) relief learning (Berridge, 2000). The state of increased motivation in the test condition might induce the release of dopamine (c.f. Becker et al., 2013), possibly leading to the release of endogenous opioids (Morgan and Franklin, 1990) when the subject won in the wheel of fortune game, which might be the neurochemical underpinning of the observed endogenous pain inhibition. Although the Motivation--‐Decision (MD) model does not explicitly differentiate the processes of wanting and liking, these considerations are in line with the model, which states that in a situation in which pain and reward are simultaneously present and reward is prioritized, pain inhibition occurs via endogenous opioidergic mechanisms. The MD model does not detail any neurophysiological mechanism of prioritizing motivations, but suggests the involvement of mesolimbic (dopaminergic) systems. We have amended the introduction and the discussion to clarify the difference between pain ratings as an indicator of (relief) liking and motivation.

Query 6: Sixth, is it really fair to call the effect an intrinsic reward, because in psychology, this is normally used to refer to evolutionarily shaped incentives such as play, exploration, novelty seeking etc.?

Response: Because the term "intrinsic reward" has been used differently in different contexts, we decided to avoid this term throughout the manuscript. We have replaced "intrinsic reward" by the descriptive terms "pain relief as the offset of a negative stimulus associated with reward" (page 3, lines 49--‐50) and "pain relief as reward " (page 3, lines 53).

Minor points

Query 7: The name of the institution which approved the study should be specified.

Response: The name of the institution that approved the study was not included in the present form of the manuscript because this was requested by the regulations of journal to keep anonymity. The name of the institution will be included in the final version of the manuscript, if accepted.

Query 8: Does the loss negative result rule out controllability relief? If so, the authors may want to perform an interaction analysis to show it?

Response:

Controllability of pain has been shown to result in decreased pain perception. It is therefore correct that the lack of a pain enhancing effect in the test condition in which participants lost could be caused by an increased sense of control compared to the control condition. However, we think this is highly unlikely because if a sense of control did play a role in the employed wheel of fortune task, pain should be less in the test compared to the control condition when no change of stimulus intensity occurred. No difference between test and control trials was observed for the 'no change' outcome for the behaviorally assessed pain perception. Subjective pain ratings where even higher in the test condition compared to the control condition for the 'no change' outcome, i.e. opposite to what would be expected if controllability did play a role. We added a statement in the discussion to highlight that controllability cannot explain the present results (page 20, lines 444--‐453).

Query 9: Does the study have any implications for the site of inhibition (cortical/spinal)?

Response: The present data does not allow any conclusions on the possible site of inhibition. Due to a lack of studies using similar paradigms that would allow speculating w.r.t. the site of inhibition, we do not include this aspect in the Discussion.

Query 10: It should be specified by how much the probabilities that were actually realized in the experiment differed from the stated probabilities of 1/3.

Response: A pseudorandomized order of the trials was used by which it was ensured that all outcomes occurred with the same probability of 1/3 (within the test and control conditions). To clarify this point, we added the following sentence "Therefore, across the test and the control trials each outcome (pain relief, pain increase, no change) occurred with a fixed probability of 1/3." (page 8, lines 170--‐171).

Query 11: The authors should explain how sensitization (e.g. line 154) differs from pain relief.

Response: Sensitization describes the process of temporal summation by which continuous stimulation longer than a few seconds or repeated stimulation with the same stimulation intensity is gradually perceived as more intense. In contrast, pain relief describes the removal of painful stimulation (partly or complete). To measure the occurrence of sensitization, we used a discrimination task, in which participants down--‐regulated the applied stimulus intensity if they perceived an increase in their sensation (and up--‐regulated the temperature if they perceived a decrease in their sensation, i.e. if they habituated). Thereby, we obtained behavioral measures of pain sensitization and habituation (c.f. Kleinböhl et al., 1999; Hölzl et al., 2005). It is thinkable that the down--‐regulation of the temperature led to pain relief. However, if such pain relief was induced by the down--‐regulation it occurred in both conditions, i.e. when participants played the game vs. when they did not play the game. Because the results are based on the comparison of these two conditions, they cannot be confounded by pain relief possibly induced by down--‐regulation of the temperature.

Query 12: It should be clarified whether the relation of pain--‐inhibition with novelty seeking arises primarily from test trials or control trials by computing separate correlations.

Response: Computing separate correlations shows that the relation of pain inhibition with novelty seeking is neither predominately driven by the test trials (r=--‐0.15, p=0.48) nor the control trials (r=0.08, p=0.72). This indicates that novelty seeking is specifically related to the induced pain inhibition linked to winning pain relief and not to the level of the perceived pain in either condition. We added this result in the results section for clarification (page 14, lines 310--‐314).

Additional concerns of a third reviewer

Query 13: Poorly controlled temperature durations leading to uncontrolled habituation/sensitization confounds.

Response: Based on the reviewer's comment, we tested whether the durations of the trials differed in the different conditions of the task. Because they were not different (mixed model ANOVA, interaction 'outcome' x 'trial type' F150 = 0.23, p = 0.80; all post--‐hoc comparisons p > 0.25), we conclude that the variable stimulation durations did not confound the results. To clarify this point we added this result in on pages 11--‐12, lines 253--‐356.

Query 14: Attention/distraction not considered really.

Response: Effects of attention or distraction should be particularly prominent in the 'no change' outcome. As discussed above in response to Query 8, behaviorally assessed pain did not differ between test and control trials for the 'no change' outcome and pain ratings were higher in the test condition in the 'no change' outcome. Because this is opposite to the well--‐described effects of distraction on pain, we are confident that distraction effects cannot explain our finding that pain relief linked to winning induces endogenous pain inhibition. The possible effects of distraction are discussion on page 20, lines 441--‐453.

Query 15: Not linking the choice to the outcome surely messes up sense of reward despite their argument about limiting learning

Response: If the wheel landed on the color the participant chose, he/she won a temperature decrease. By linking the outcome to luck, learning a favorable strategy for color selection is excluded. Despite the limited learning, a vast amount of literature on gambling shows that reward, even if only vaguely and not controllably linked to choice behavior, induces a strong sense of reward (e.g. winning in a slot machine game). Reward obtained by gambling induces very positive feelings, in line with the well described phasic dopaminergic responses that are much higher with unexpected than with expected rewards (Fiorillo et al., 2003). The finding that playing for the relief induced endogenous pain inhibition indicates that the task clearly had effects that were not observed in the control condition. Likely, these effects are related to activation of relief/reward systems in the brain albeit we cannot prove this with the present study.

Query 16: Not convinced that losing and neutral are really both control conditions.

Response: The goal of the present study was to investigate the effects of winning a temperature decrease in a condition comprising motivated behavior. The main control condition of the experiment was the condition in which participants received the same temperature decrease for which they had not played. In order to establish that the engagement of endogenous pain inhibition was indeed due to having won the temperature decrease and not to unspecific effects of playing the game, such as distraction or sense of control, we included two outcomes that controlled for different variables. First, a 'no change' outcome in which the temperature did not change. Second, a temperature increase that was coupled to having lost. The 'no change' outcome served to control for any unspecific effects for which the difference between test and control trials should not differ for the pain relief and the 'no change' outcomes, such as distraction. In addition, the losing outcome was included because winning has been associated with arousal, which we hypothesized would not be present in the neutral 'no change' outcome. Losing is similarly associated with arousal (Sokol--‐Hessner et al., 2009; 2013) and therefore ensured that any finding regarding winning is not simply caused by arousal. To clarify this point we added this result in on page 6, lines 136--‐138.

Query 17: Memory confounds in recalibration of memorised temp not assessed so not a reliable method.

Response: The discrimination task operationalized by the self--‐adjustment of the stimulation intensity to investigate sensitization and habituation has been established and validated before (Kleinböhl et al., 1999; 2006). Although we cannot exclude that memory confounds might affect this task, such effects should have occurred in all trials. Therefore, memory confounds cannot explain the difference between test and control trials.

References

Becker S, Gandhi W, Elfassy NM, Schweinhardt P (2013) The role of dopamine in the perceptual modulation of nociceptive stimuli by monetary wins or losses. Eur J Neurosci 38:3080-3088.

Becker S, Kleinböhl D, Hölzl R (2012) Awareness is awareness is awareness? Decomposing different aspects of awareness and their role in operant learning of pain sensitivity. Consciousness and cognition 21:1073-1084 Available at: http://www.ncbi.nlm.nih.gov/entrez/query.fcgi?cmd=Retrieve&amp;db=PubMed&amp;dopt=Citation&amp;list_uids=22521471.

Berridge KC (2000) Reward learning: Reinforcement, incentives, and expectations. Psychology of learning and motivation.

Clark L, Lawrence AJ, Astley--‐Jones F, Gray N (2009) Gambling near--‐misses enhance motivation to gamble and recruit win--‐related brain circuitry. Neuron 61:481-490.

Dong G, Lin X, Zhou H, Lu Q (2014) How the win--‐lose balance situation affects subsequent decision--‐ making: functional magnetic resonance imaging evidence from a gambling task. Neuroscience 272:131-140.

Fiorillo CD, Tobler PN, Schultz W (2003) Discrete coding of reward probability and uncertainty by dopamine neurons. Science 299:1898-1902.

Gerber B, Yarali A, Diegelmann S, Wotjak CT, Pauli P, Fendt M (2014) Pain--‐relief learning in flies, rats, and man: basic research and applied perspectives. Learn Mem 21:232-252.

Hölzl R, Kleinböhl D, Huse E (2005) Implicit operant learning of pain sensitization. Pain 115:12-20.

Kleinböhl D, Gortelmeyer R, Bender HJ, Hölzl R (2006) Amantadine sulfate reduces experimental sensitization and pain in chronic back pain patients. Anesth Analg 102:840-847.

Kleinböhl D, Hölzl R, Möltner A, Rommel C, Weber C, Osswald PM (1999) Psychophysical measures of sensitization to tonic heat discriminate chronic pain patients. Pain 81:35-43.

Loeser JD, Treede RD (2008) The Kyoto protocol of IASP Basic Pain Terminology. Pain 137:473-477.

Martinez F, Bonnefon J--‐F, Hoskens J (2009) Active involvement, not illusory control, increases risk taking in a gambling game. Q J Exp Psychol (Hove) 62:1063-1071.

Morgan MJ, Franklin KB (1990) 6--‐Hydroxydopamine lesions of the ventral tegmentum abolish D--‐ amphetamine and morphine analgesia in the formalin test but not in the tail flick test. Brain Res 519:144-149.

Sokol--‐Hessner P, Camerer CF, Phelps EA (2013) Emotion regulation reduces loss aversion and decreases amygdala responses to losses. Soc Cogn Affect Neurosci 8:341-350.

Sokol--‐Hessner P, Hsu M, Curley NG, Delgado MR, Camerer CF, Phelps EA (2009) Thinking like a trader selectively reduces individuals' loss aversion. Proc Natl Acad Sci USA 106:5035-5040.
